# Endothelial cell-specific reduction of heparan sulfate suppresses glioma growth in mice

**DOI:** 10.1007/s12672-021-00444-3

**Published:** 2021-11-11

**Authors:** Takamasa Kinoshita, Hiroyuki Tomita, Hideshi Okada, Ayumi Niwa, Fuminori Hyodo, Tomohiro Kanayama, Mikiko Matsuo, Yuko Imaizumi, Takahiro Kuroda, Yuichiro Hatano, Masafumi Miyai, Yusuke Egashira, Yukiko Enomoto, Noriyuki Nakayama, Shigeyuki Sugie, Kazu Matsumoto, Yu Yamaguchi, Masayuki Matsuo, Hideaki Hara, Toru Iwama, Akira Hara

**Affiliations:** 1 Department of Tumor Pathology, Gifu University Graduate School of Medicine, Gifu, 501-1194 Japan; 2 Department of Neurosurgery, Gifu University Graduate School of Medicine, Gifu, 501-1194 Japan; 3 Department of Emergency and Disaster Medicine, Gifu University Graduate School of Medicine, Gifu, 501-1194 Japan; 4 Department of Radiology, Gifu University Graduate School of Medicine, Gifu, 501-1194 Japan; 5 Department of Neurosurgery, Ogaki Tokusyukai Hospital, Ogaki, Gifu 503-0015 Japan; 6 Department of Pathology, Asahi University Hospital, Gifu, 500-8523 Japan; 7 Department of Orthopaedic Surgery, Gifu University Graduate School of Medicine, Gifu, 501-1194 Japan; 8 Sanford Burnham Prebys Medical Discovery Institute, La Jolla, San Diego, CA USA; 9 Molecular Pharmacology, Department of Biofunctional Evaluation, Gifu Pharmaceutical University, Gifu, 501-1196 Japan

**Keywords:** Glioblastoma, Angiogenesis, Heparan sulfate, Fibroblast growth factor 2

## Abstract

**Purpose:**

Heparan sulfate (HS) is one of the factors that has been suggested to be associated with angiogenesis and invasion of glioblastoma (GBM), an aggressive and fast-growing brain tumor. However, it remains unclear how HS of endothelial cells is involved in angiogenesis in glioblastoma and its prognosis. Thus, we investigated the effect of endothelial cell HS on GBM development.

**Methods:**

We generated endothelial cell-specific knockout of *Ext1*, a gene encoding a glycosyltransferase and essential for HS synthesis, and murine GL261 glioblastoma cells were orthotopically transplanted. Two weeks after transplantation, we examined the tumor progression and underlying mechanisms.

**Results:**

The endothelial cell-specific *Ext1* knockout (*Ext1*^*CKO*^) mice exhibited reduced HS expression specifically in the vascular endothelium of the brain capillaries compared with the control wild-type (WT) mice. GBM growth was significantly suppressed in *Ext1*^*CKO*^ mice compared with that in WT mice. After GBM transplantation, the survival rate was significantly higher in *Ext1*^*CKO*^ mice than in WT mice. We investigated how the effect of fibroblast growth factor 2 (FGF2), which is known as an angiogenesis-promoting factor, differs between *Ext1*^*CKO*^ and WT mice by using an in vivo Matrigel assay and demonstrated that endothelial cell-specific HS reduction attenuated the effect of FGF2 on angiogenesis.

**Conclusions:**

HS reduction in the vascular endothelium of the brain suppressed GBM growth and neovascularization in mice.

**Supplementary Information:**

The online version contains supplementary material available at 10.1007/s12672-021-00444-3.

## Introduction

Glioblastoma (GBM) is one of the most aggressive and common tumors of the central nervous system and is characterized by strong infiltration and angiogenesis [1, 2]. Despite current combination therapies such as surgical resection, radiation therapy, and chemotherapy, the prognosis for GBM patients remains poor; most patients still have a median survival of less than 15 months, and the 5 years survival rate is less than 3% [3].

Angiogenesis is a prominent feature of glioblastoma, and various approaches have been used to control it. The anti-vascular endothelial growth factor (VEGF) humanized monoclonal antibody bevacizumab has shown an improvement in progression-free survival (PFS) in combination therapy [4, 5]. However, there is no improvement in overall survival, and there is an urgent need to develop an approach to improve the prognosis of GBM.

Heparan sulfate proteoglycans (HSPGs), a group of glycoconjugates composed of heparan sulfate (HS) chains covalently attached to core protein, are present on the cell surface and extracellular matrix and are required for the activity of angiogenic factors, such as vascular endothelial growth factor A (VEGFA) and fibroblast growth factor 2 (FGF2) [6–8]. There have been reports that inhibition of FGF2 and its receptor signaling suppresses the proliferation of GBM cells, and that HSPGs in GBM promote tumor invasion [9, 10]. Thus, HSPGs have been suggested to have significant effects on angiogenesis and GBM invasion; however, it is still unclear how HS in the intratumoral vascular endothelium affects GBM progression. The synthesis of the HS backbone is mediated by glycosyltransferases of the exostosin family, including Ext1, Ext2, and others [11]. In particular, Ext1 is essential for the synthesis of HS. Cells lacking the functional *Ext1* allele do not synthesize HS, and *Ext1* knockout mice die during early embryogenesis due to gastrulation defects [12, 13].

In this study, we examined how HS-reduced vascular endothelial cells are involved in the growth of glioma by using mice in which *Ext1* expression is specifically ablated in vascular endothelial cells.

## Materials and methods

### Mice

Six-week-old C57BL6/J mice were purchased from Charles River Japan (Kanagawa, Japan). VE-Cadherin-Cre mice were purchased from the Laboratory Animal Resource Bank at NIBIOHN [14] and *Rosa26‐CAG‐LSL‐tdTomato* (Ai9, Jax#: 007905) mice were purchased from Jackson Laboratories (Bar Harbor, ME, USA). The mouse *Ext1*^*flox*^ allele was described previously [12]. Male and female mice were bred, maintained, and housed in an air-conditioned room (23 ± 2 °C) with a 12 h light/dark cycle. These mice were provided free access to food and tap water.

### Cell lines and culture

GL261 glioma cells were kindly provided by Dr. Masanao Saio (Gunma University, Japan) and were cultured in Dulbecco’s modified Eagle’s medium (DMEM) supplemented with 10% fetal bovine serum and 0.1% penicillin–streptomycin solution at 37 °C and 5% CO_2_. All experiments were performed within 10 passages of the frozen stock.

### Intracranial tumor establishment

For intracranial glioma cell implantation, 1.0 × 10^5^ cells were suspended in 2 μL phosphate-buffered saline (PBS). Briefly, 8–12-week-old C57BL/6 mice were anesthetized using three types of mixed intraperitoneal administration anesthesia reported previously by Kawai et al. [15], and stereotactically injected using a 26-gauge Hamilton microliter syringe into the brain at the following coordinates: 2 mm lateral and 1 mm anterior to bregma at a depth of 3 mm. After waiting for 1 min, the cell solution (2 µL) was added at a rate of 1 µL/min. The mice were then allowed to rest for 1 min, and the needle was pulled out as slowly as possible.

### Tissue preparation

After anesthesia, the thorax of the mice was opened, and the inferior vena cava was incised. Perfusion washing using a drip infusion system was performed with equal volumes of cold 0.1 M PBS and cold 4% paraformaldehyde (PFA) solution. The brains were dissected, divided into pieces, and used for preparing paraffin and frozen sections.

### Histological and immunohistochemical procedures

Paraffin blocks were cut into 3 µm thick sections and subjected to hematoxylin and eosin (H&E) staining as routine procedures. Adjacent serial sections were subjected to immunohistochemistry for FGF2, Ki67, and Iba1. For immunostaining, deparaffinized sections were subjected to autoclave boiling in 0.015 M sodium citrate buffer solution (pH 6.0) for 10 min at 110 °C as an antigen retrieval procedure before incubation with 3% H_2_O_2_ diluted in methanol for 10 min and blocked with 2% normal bovine serum.

Sections were incubated with rabbit anti-FGF2 antibody (dilution 1:200, Bioss), rabbit anti-Ki67 antibody (dilution 1:500, CST), rabbit anti-Iba1 antibody (dilution 1:500, Wako), rabbit anti-phospho-ERK1/2 (pERK1/2) antibody (dilution 1:200, CST), or rabbit anti-ERK1/2 antibody (dilution 1:100, CST) overnight at 4 °C, followed by incubation with conjugated secondary antibodies for 60 min at 37 °C. Immunoreaction was visualized using 3,3′-diaminobenzidine tetrahydrochloride (DAB, Sigma). The sections were counterstained with hematoxylin. Fluorescent staining was used for rat anti-CD31 antibody (dilution 1:50, Dianova) and rabbit anti-Red Fluorescent Protein (RFP) antibody (dilution 1:500, Rockland) double staining with fluorophore-conjugated secondary antibodies for 60 min at 37 °C. Anti-RFP antibody was used to identify the location of tdTomato in paraffin sections.

Frozen sections were used for lectin and dextran staining. Fifteen minutes after injecting lectin (50 µg/100 µl dH_2_O, VECTOR) or dextran (2000 kDa, 200 µg/100 µl dH_2_O, Sigma) via the jugular vein, the brain was removed and fixed in 4% paraformaldehyde overnight at 4 °C, and then incubated in 30% sucrose for cryoprotection (4 °C for 2–3 days), embedded in the optimal cutting temperature (OCT) compound, and frozen with liquid nitrogen. They were sectioned coronally at 5 µm with a cryostat (Leica), and each section was stored at − 80 °C. For immunostaining, sections were incubated with rabbit anti-PDGFRβ antibody (dilution 1:500, Abcam) or mouse anti-HS antibody (clone F58-10E4, dilution 1:100, Amsbio) overnight at 4 °C, followed by incubation with conjugated secondary antibody for 60 min at 37 °C.

### Immunostaining of cells

Endothelial cells were isolated from the mouse brain using CD31 microbeads (Miltenyi Biotec). Cells were fixed with a 15-min incubation in 4% PFA. After washing thrice with PBS, the cells were permeabilized in 0.2% Tween solution in PBS for 15 min. Cells were washed three times in PBS, blocked with 2% bovine serum in PBS for 1 h, and subsequently stained with rabbit anti-Ext1 antibody (dilution 1:50, Bioworld) or mouse anti-HS antibody (dilution 1:100, Amsbio) overnight at 4 °C. Cells were washed thrice with PBS, incubated with fluorophore-conjugated secondary antibodies for 1 h at room temperature, and finally stained with DAPI (dilution 1:1000, Wako).

### Matrigel angiogenesis assay

*Ext1*^*flox/flox*^*; VE-Cre*; *Lsl-tdTomato* and control (*VE-Cre*; *Lsl-tdTomato)* mice were injected subcutaneously at the lateral abdominal area with 0.5 ml of Matrigel premixed on ice with 200 ng/2 µl recombinant mouse FGF2 (Abcam) or PBS as control. After 2 weeks, Matrigel was removed and fixed in 4% paraformaldehyde at room temperature for 4 h and then incubated in 20% sucrose for cryoprotection, at room temperature for 18 h, embedded in the OCT compound, and frozen with liquid nitrogen. They were sectioned coronally at 14 µm with a cryostat (Leica), and each section was stored at − 80 °C. For immunostaining, Matrigel sections were mounted onto frosted microscope slides and blocked with 2% bovine serum in PBS for 1 h. Tissue sections were then stained with rat anti-CD31 antibody (dilution 1:50, Dianova) and incubated overnight at 4 °C. Next, the tissue was washed thrice, incubated with fluorophore-conjugated secondary antibodies for 1 h at room temperature, and finally stained with DAPI (dilution 1:1000, Wako).

### Real-time RT-PCR

Total RNA was isolated from transplanted tumors or endothelial cells from the mouse brain using the Maxwell RSC simplyRNA Tissue Kit (Promega Corp.). cDNA was synthesized using the SuperScript III First-Strand Synthesis Kit (Life Technologies). Quantitative real-time RT-PCR was performed using the StepOnePlus system (Applied Biosystems). The primers used for real-time RT-PCR are listed in Supplementary Table 1. The comparative Ct method was used to analyze the relative gene expression. Two independent experiments were performed in duplicate in each experiment. Beta-actin was used as a housekeeping gene for normalization.

### Microarray analysis

Total RNA was isolated from transplanted tumors using the Maxwell RSC simplyRNA Tissue Kit (Promega Corp.). Cy3-labeled probes were prepared from total RNA using the Low Input Quick Amp Labeling Kit 1-color (Agilent Technologies) and hybridized with a microarray slide (SurePrint G3 Mouse GE 8 × 60 K Microarray; Agilent Technologies) for 17 h at 65 °C. Next, the slide was washed and scanned with a microarray scanner (ArrayScan, Agilent Technologies) to obtain the probe’s fluorescent signal, processed for digitization using Feature Extraction software (Agilent Technologies), and analyzed with GeneSpring GX software (Agilent Technologies) for gene expression analysis. Moderated* t*-tests were used to reveal significant differences in gene expression between tumors in control and *Ext *^*flox/flox*^*; VE-Cre* mice.

### Statistical analysis

For mouse experiments, statistical details of the experiments can be found in the figure legends. Statistical significance was determined using Student’s *t*-test, Welch’s *t*-test, Tukey’s test, or log-rank test. All statistical analyses were performed using JMP 14.2 (SAS Institute Inc.). Statistical significance was set at P ≤ 0.05 for all analyses.

## Results

### HS reduction in endothelial cell-specific Ext1 knockout mice

To examine whether HS on vascular endothelial cells of the brain affects endothelial homeostasis, we generated epithelial cell-specific *Ext1* knockout mice by crossing *Ext1*^*flox/flox*^ with VE-cadherin Cre/ + mice (*Ext1*^*flox/flox*^*;VE-cadherin-Cre*). *Ext1* encodes a glycosyltransferase that catalyzes the polymerization of alternating glucuronic acid and *N*-acetylglucosamine sugar residues in the HS biosynthetic process [16]. Ext1 is indispensable for HS synthesis, as cells lacking a functional *Ext1* allele cannot synthesize HS [17, 18]. Endothelial cell-specific *Ext1* knockout mice are referred to as *Ext1*^*CKO*^ mice.

**Fig. 1:**
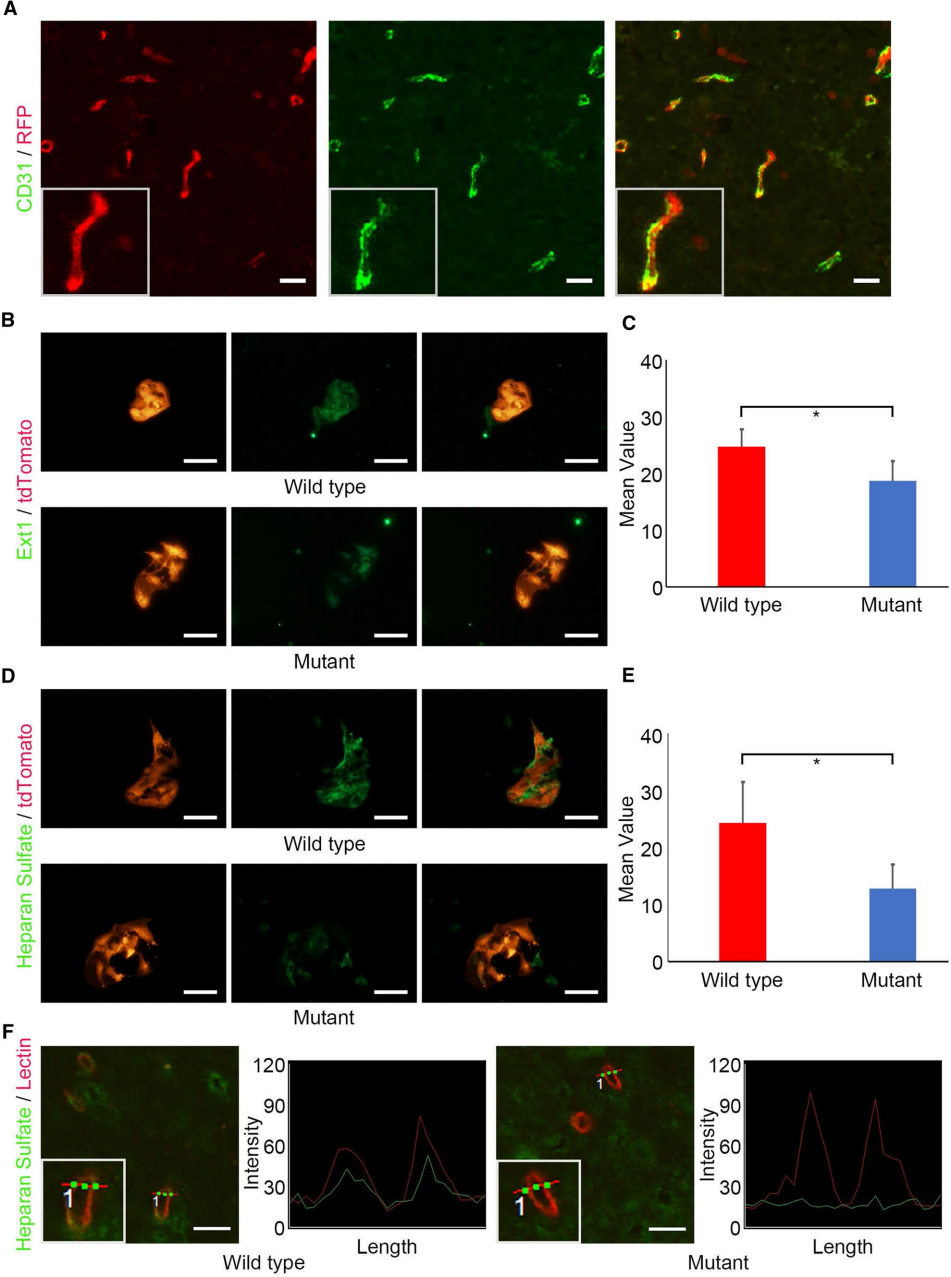
Specific reduction of heparan sulfate in endothelial cells of murine brain. **a** Fluorescent double staining of CD31 and RFP in murine brains of *Ext1*^*flox/flox*^*; VE-Cre*; *Lsl-tdTomato* and control (*VE-Cre*; *Lsl-tdTomato)* mice. Scale bar = 100 µm. **b**–**e** Ext-1 (**b**) or heparan sulfate (**d**) expressions of isolated endothelial cells from the brains of *Ext1 *^*flox/flox*^*; VE-Cre*; *Lsl-tdTomato* and control (*VE-Cre*; *Lsl-tdTomato)* mice. Quantifications of Ext-1 (**c**) or heparan sulfate (**e**) expressions of isolated endothelial cells from the brains of *Ext1*^*flox/flox*^*; VE-Cre*; *Lsl-tdTomato* and control (*VE-Cre*; *Lsl-tdTomato)* mice. (n = 5 each cohort. Bars represent the mean ± SD. Student *t*-test, *P < 0.05) Scale bar = 50 µm. f. Endothelial heparan sulfate expression and its intensity graph of capillary lumens in the brains of *Ext1*^*flox/flox*^*; VE-Cre* and control (*Ext1 *^*flox/flox*^* or Ext1*^*flox/*+^*)* mice. *Insets indicate high magnification of brain blood vessel.* Scale bar = 50 µm

By crossing *VE-cadherin-Cre* with *Lsl-tdTomato* reporter mice, we confirmed that Cre recombinase under the VE-cadherin promoter was almost activated in vascular endothelial cells with CD31 expression (Fig. 1a). Next, we isolated vascular endothelial cells from *Ext1*^*CKO*^; *Lsl-tdTomato,* and control (*VE-cadherin-Cre*; *Lsl-tdTomato)* mice and examined the Ext1 and HS expression by immunofluorescent staining (Fig. 1b–e). Ext1 expression in endothelial cells of *Ext1*^*CKO*^*; Lsl-tdTomato* mice was significantly lost compared with that of control mice, although small cross reactivity was observed (Fig. 1b and c). Similarly, HS expression in endothelial cells of *Ext1*^*CKO*^*; Lsl-tdTomato* mice was significantly reduced compared with that of control mice (Fig. 1d and e). This is consistent with previous reports that Ext2 may rescue HS synthesis in vivo or in vitro to some extent [11, 19, 20]. In our study, *Ext2* gene expression level was not significantly reduced, so Ext2 may rescued HS synthesis as in previous reports (Fig. S1a). Because the blood vessels genetically modified did not regenerate so much in the Matrigel assay, it was also suggested that the blood vessels with a large decrease in heparan sulfate may not be able to grow properly (Fig. S2a).

To assess the ablation of HS expression in the endothelium in the *Ext1*^*CKO*^ mouse brain, we examined HS expression of the vascular endothelium by using anti-HS antibody with an intravenous injection of lectin to detect vascular endothelium. Immunofluorescent staining revealed that HS expression in the lumen of capillaries was reduced in *Ext1*^*CKO*^ mice compared with that in control mice (Fig. 1f). These results indicate that endothelial cell-specific ablation of *Ext1* leads to reduced HS in the brain capillaries of mice.

### HS reduction doesn’t affect the homeostasis of capillary function in vivo

**Fig. 2:**
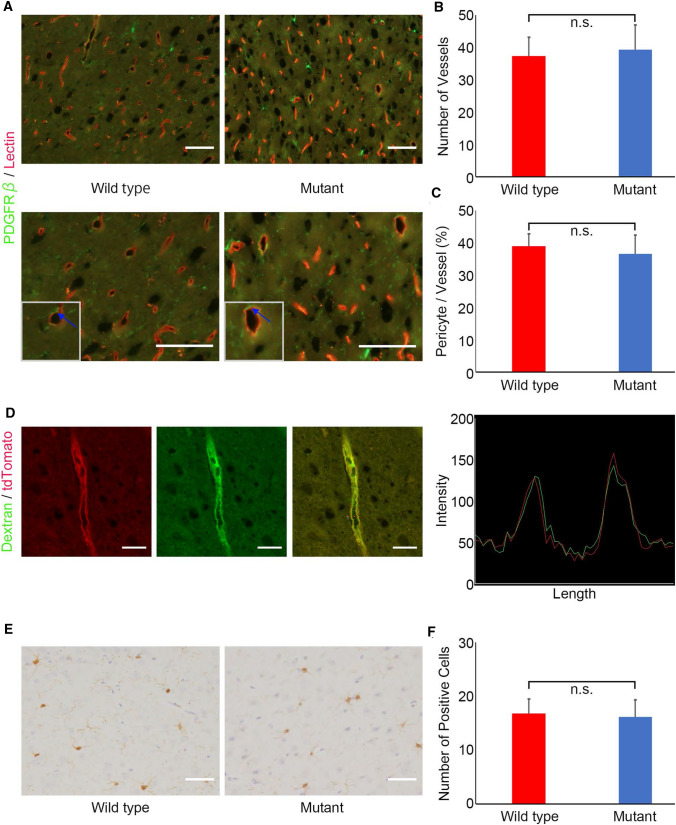
No significant differences in the homeostasis of capillary function with heparan sulfate reduction. **a** Pericytes adjacent to capillaries evaluated by fluorescent immunostaining with anti-PDGFRβ antibody in the brain of *Ext1*^*flox/flox*^*; VE-Cre* and control (*Ext1*^*flox/*+^*)* mice. Lower shows a more strongly enlarged figure. *Arrows in insets indicate high magnification of pericytes.* Scale bar = 50 µm. **b** Quantification of brain blood vessels of in *Ext1*^*flox/flox*^*; VE-Cre* and control (*Ext1*^*flox/*+^*)* mice. (n = 5, each cohort. Bars represent the mean ± SD. Student *t* test, n.s. = not significant). **c** Quantification of pericyte proportion in brain capillaries of in *Ext1 *^*flox/flox*^*; VE-Cre* and control (*Ext1*^*flox/*+^*)* mice. (n = 5, each cohort. Bars represent the mean ± SD. Student *t*-test, n.s. = not significant). **d** Vascular permeability and its intensity graph in brain capillaries of *Ext1 *^*flox/flox*^*; VE-Cre*; *Lsl-tdTomato* and control (*VE-Cre*; *Lsl-tdTomato)* mice. Dextran (green), FITC-Dextran (2000 kDa). Scale bar = 100 µm. **e** Iba1 (microglia marker) expression in brain capillaries of *Ext1 *^*flox/flox*^*; VE-Cre* and control (*Ext1 *^*flox/flox*^* or Ext1*^*flox/*+^*)* mice. Scale bar = 50 µm. **f** Quantification of Iba1 positive cells in brain capillaries of *Ext1 *^*flox/flox*^*; VE-Cre* and control (*Ext1*^*flox/flox*^* or Ext1*^*flox/*+^*)* mice. (n = 10 each cohort. Bars represent the mean ± SD. Student *t*-test, n.s. = not significant)

While we observed *Ext1*^*CKO*^ mice for approximately a year carefully, *Ext1*^*CKO*^ mice did not show any abnormalities or diseases other than a slightly smaller body compared with control mice. To investigate the effect on the blood–brain barrier (BBB) in endothelial cell-specific HS reduction, we examined the relationship between endothelial cells and pericytes, one of the constituents of the BBB and blood vessels in the brain. Immunofluorescent staining using anti-PDGFRβ antibody, one of the markers for pericytes, showed no significant difference in the number of blood vessels and the ratio of pericytes around the blood vessels to the number of blood vessels between the two cohorts (Fig. 2a–c).

To evaluate vascular permeability in the BBB, we administered fluorescent dextran. No extravasation was observed in *Ext1*^*CKO*^ mice, suggesting that the function, at least in vascular permeability, may be maintained (Fig. 2d). Changes in microglia were also examined using Iba1 immunostaining as an evaluation of the immune system, but no significant changes were observed between the two cohorts (Fig. 2e, f). These data demonstrated that endothelial cell-specific HS reduction may not affect, in a part, the murine BBB homeostasis, which is associated with vascular permeability.

### HS reduction in the endothelium of brain capillaries suppressed glioma growth in vivo

**Fig. 3:**
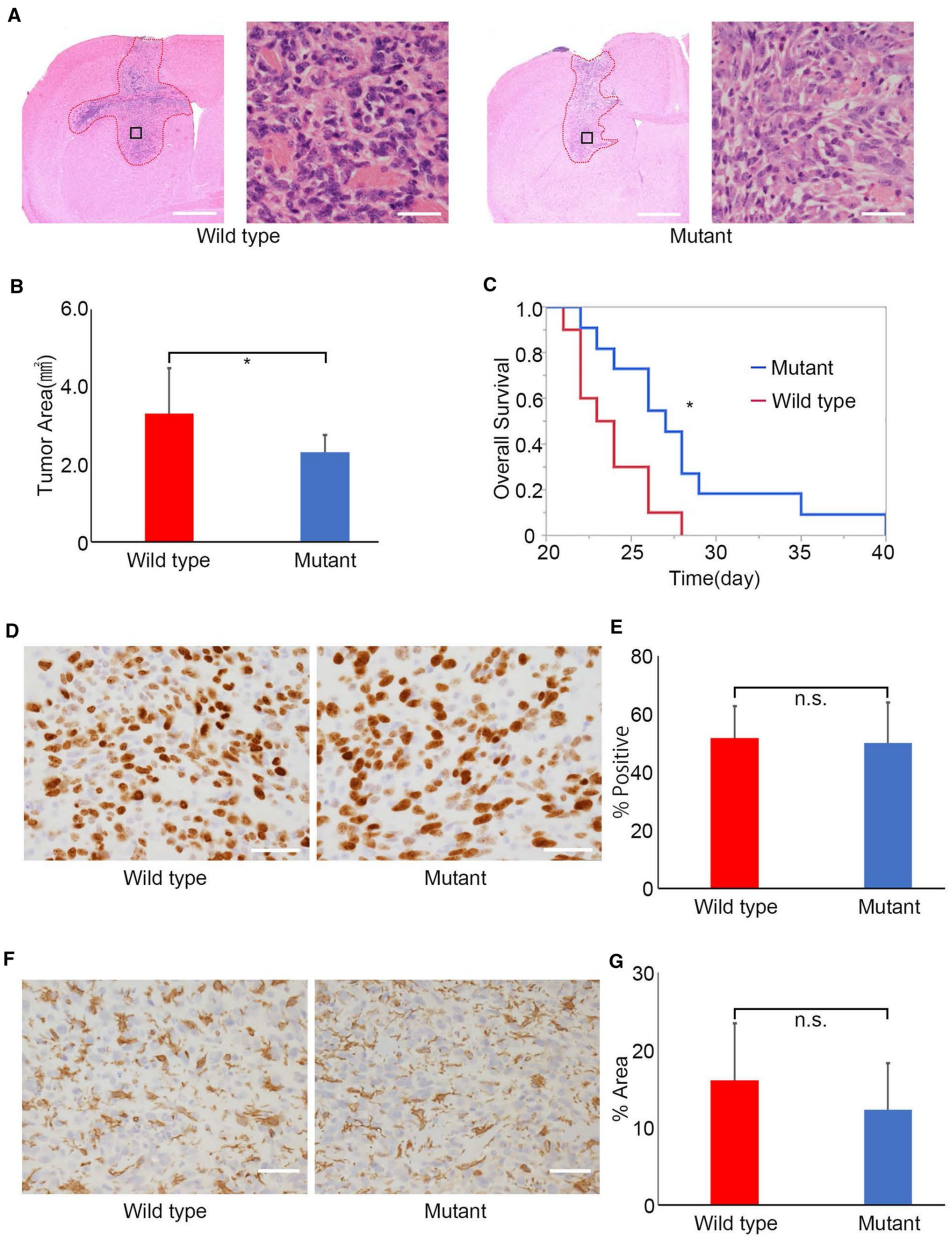
Heparan sulfate reduction in brain capillaries suppresses GL261 glioma growth in vivo. **a** Representative H&E staining of murine brains of *Ext1*^*flox/flox*^*; VE-Cre* and control (*Ext1*^*flox/flox*^* or Ext1*^*flox/*+^*)* mice following transplantation of GL261 glioma cells. Scale bar = 1 mm and 50 µm, respectively. **b** Tumor area quantification in brain tumors of *Ext1*^*flox/flox*^*; VE-Cre* and control (*Ext1*^*flox/flox*^* or Ext1*^*flox/*+^*)* mice. (n = 11 and 10, respectively. Bars represent the mean ± SD. Welch *t*-test, *P < 0.05). **c** Overall survival of *Ext1*^*flox/flox*^*; VE-Cre* and control (*Ext1*^*flox/flox*^* or Ext1*^*flox/*+^ *or Ext1*^+*/*+^*)* mice. (n = 10 and 11, respectively. log-rank test, *P < 0.05). **d**–**g** Immunostaining of Ki67 (**d**) and Iba1 (**f**) in tumor tissues of *Ext1*^*flox/flox*^*; VE-Cre* and control (*Ext1*^*flox/flox*^* or Ext1*^*flox/*+^*)* mice. Quantification of Ki67 (**e**) and Iba1 (**g**) in tumor tissues of *Ext1*^*flox/flox*^*; VE-Cre* and control (*Ext1*^*flox/flox*^* or Ext1*^*flox/*+^*)* mice. Scale bar = 50 µm (n = 9 and 10, respectively. Bars represent the mean ± SD. Student *t* test, n.s. = not significant)

To test whether endothelial cell-specific HS reduction affects tumor growth in the murine brain, we performed an orthotopic transplantation experiment with murine glioma GL261 cells. Fourteen days after the tumor was transplanted, we performed brain excision to evaluate the tumor transplant site histologically by H&E staining according to the previous report [21] (Fig. 3a, Fig. S3a, b). The tumor area at the tumor transplantation site was significantly reduced in *Ext1*^*CKO*^ mice compared with that in control mice (Fig. 3b). We next confirmed whether there was a change in the overall survival time. As a result, a significant increase in survival time was observed in *Ext1*^*CKO*^ mice compared to control mice (Fig. 3c).

To elucidate the mechanisms underlying the difference in tumor volume between *Ext1*^*CKO*^ and control mice, we performed immunostaining for Ki67, a proliferation marker, in both cohorts. The results showed no significant difference between the two cohorts (Fig. 3d, e). Furthermore, intratumoral immunity and microglial infiltration were evaluated by immunostaining for Iba1, a microglia marker; however, there was no significant change in the Iba1-positive cell area between the two cohorts (Fig. 3f, g). These results indicate that endothelial cell-specific HS reduction, at least, suppresses glioma growth and prolongs overall survival.

### HS reduction resulted in intratumoral angiogenesis suppression in the glioma microenvironment

**Fig. 4:**
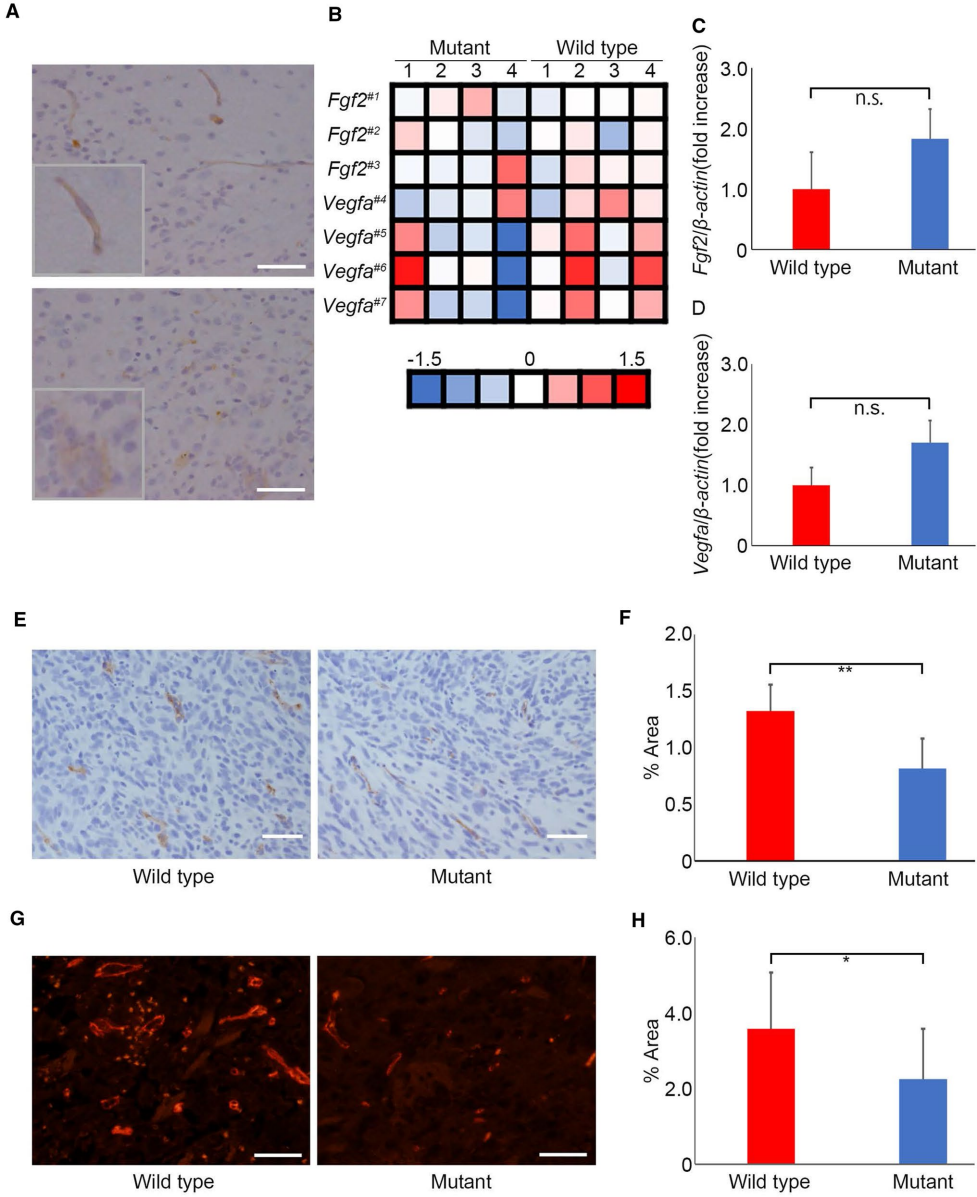
Intratumoral angiogenesis is inhibited by heparan sulfate reduction in brain capillaries. **a** Immunostaining of FGF2 in brain tissues transplanted GL261 glioma cells in E*xt1*^*flox/flox*^; *VE-Cre. Insets indicate high magnification of FGF2-positive endothelial cells in capillaries (upper) and FGF2-positive tumor tissues (lower).* Scale bar = 50 µm. **b** Transcriptional profiles related to angiogenesis in transplanted GL261 glioma tissues in E*xt1*^*flox/flox*^*;VE-Cre* and control (*Ext1*^*flox/flox*^*)* mice (n = 4 each cohort). #1–7 indicate 1: A_55_P2048705, 2: A_52_P376768, 3: A_51_P399845, 4: A_55_P2741794, 5: A_52_P638895, 6: A_51_P482552, 7: A_52_P249424, respectively. **c**, **d** Quantification of relative expressions of *Fgf2* (**c**) and *Vegfa* (**d**) in transplanted GL261 glioma tissues in E*xt1*^*flox/flox*^*; VE-Cre* and control (*Ext1*^*flox/flox*^*)* mice, evaluated by real time RT-PCR. (n = 3 each cohort. Bars represent the mean ± SD. Student *t* test, n.s. = not significant). **e** Immunostaining for CD31 in brain tissues transplanted GL261 glioma cells in E*xt1 *^*flox/flox*^*; VE-Cre* and control (*Ext1*^*flox/flox*^* or Ext1*^*flox/*+^*)* mice*. Insets indicate CD31-positive endothelial cells in capillaries.* Scale bar = 50 µm. **f** Quantification of intratumoral vessel’s area in E*xt1*^*flox/flox*^*; VE-Cre* and control (*Ext1*^*flox/flox*^* or Ext1*^*flox/*+^*)* mice. (n = 7 each cohort. Bars represent the mean ± SD. Student *t*-test, **P < 0.01). **g** Immunofluorescent staining for CD31 in brain tissues transplanted GL261 glioma cells into E*xt1*^*flox/flox*^*; VE-Cre* and control (*Ext1*^*flox/flox*^* or Ext1*^*flox/*+^*)* mice*.* Scale bar = 50 µm. **h** Quantification of CD31 immunofluorescent positive area in E*xt1 *^*flox/flox*^*; VE-Cre* and control (*Ext1*^*flox/flox*^* or Ext1*^*flox/*+^*)* mice. (n = 10 each cohort. Bars represent the mean ± SD. Student *t* test, *P < 0.05)

Since HS is required for FGF2 signal activation as a receptor [7, 8], we hypothesized that the tumor suppressive effect was due to the attenuation of angiogenesis in brain capillaries. First, in *Ext1*^*CKO*^ mice, we performed immunostaining for FGF2, a protein related to angiogenesis, to investigate its localization. FGF2 was expressed not only in the vascular epithelium but also in glioma cells, although the staining was weak (Fig. 4a). In the transcriptional profiling of whole tumor tissues in *Ext1*^*CKO*^ and control mice on vascular angiogenesis, *Fgf2* and *Vegfa* expression levels did not change significantly between the two cohorts (Fig. 4b). Real-time quantitative PCR (qPCR) also showed that relative expression levels were maintained in *Ext1*^*CKO*^ mice (Fig. 4c, d). Thus, it seemed that reduced production of FGF2 and VEGFA was not occurred in *Ext1*^*CKO*^ mice. On the other hand, pERK1/2, which is a downstream signal of FGF2, was weakened by immunostaining although ERK1/2 (ERK control without phosphorylation) was present in both groups, suggesting that FGF2-ERK signaling activation might be weak in the microenvironment (Fig. S4a-b). Secondly, we performed CD31 staining to evaluate angiogenesis within the tumor. The results showed significant suppression of angiogenesis in both immunostaining and fluorescent immunostaining in *Ext1*^*CKO*^ compared with that in control (WT) mice (Fig. 4e–h, Fig. S5a). These data suggest that glioma growth inhibition might be caused by the suppression of intratumoral angiogenesis related to FGF2.

### Endothelial cell-specific HS reduction attenuated FGF2 stimulation in vivo

**Fig. 5:**
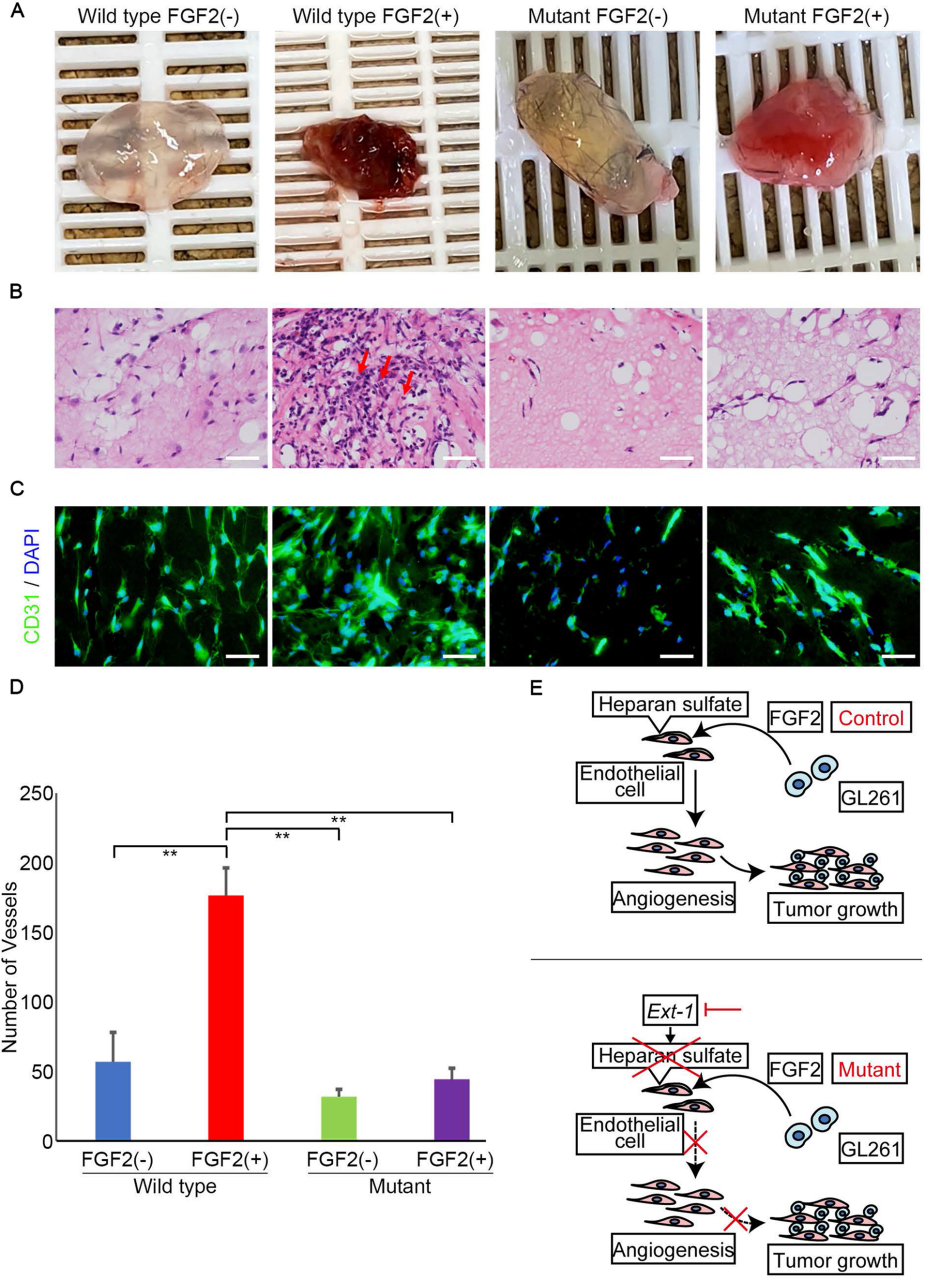
Angiogenesis induced by FGF2 stimulation is inhibited in capillaries with heparan sulfate reduction. **a** Gross appearance of Matrigel plugs resected from *Ext1*^*flox/flox*^*; VE-Cre*; *Lsl-tdTomato* and control (*VE-Cre*; *Lsl-tdTomato)* mice with or without FGF2 induction. **b**, **c** H&E staining (**b**) and CD31 immunofluorescent staining (**c**) of Matrigel plugs resected from each cohort shown in** a**.* Arrows indicate endothelial cells with inflammatory cells*. Scale bar = 50 µm. **d** Quantification of vessel number in each cohort (n = 5 each cohort. Bars represent the mean ± SD. Turkey’s test, **P < 0.01). The number is evaluated at magnifications of × 400 (high power field; HPF). **e** A schema of a mechanism found in this study

To confirm the direct role of FGF2 in angiogenesis, an in vivo angiogenesis assay was performed using Matrigel with or without mouse recombinant FGF2 protein. Matrigel with or without FGF2 was administered subcutaneously in both groups and excised 14 days later. The excised Matrigel plugs with FGF2 had a red color in appearance compared to those without FGF2 (Fig. 5a). The FGF2-containing Matrigel plug in the control group showed strong adhesion to the skin compared with that without FGF2.

In H&E staining, immune cell infiltration was observed, especially in the control group Matrigel plugs. In the FGF2-containing Matrigel plugs, blood vessels with a more solid structure were observed (Fig. 5b). CD31 fluorescent staining showed that FGF2-containing Matrigel plugs had thicker blood vessels than that without FGF2 in both groups (Fig. 5c). Evaluation of the number of endothelial cells demonstrated that the FGF2-containing Matrigel plug in the control group showed a significant increase compared with other ones (Fig. 5d). In addition, the rate of increase was larger in the control group than in the mutant group, suggesting that the effect of FGF2 was attenuated in the mutant group.

## Discussion

Here, we found that specific reduction of HS in vascular endothelial cells suppresses GBM growth in endothelial cell-specific *Ext1* knockout mice. While there are many reports that HS upregulation in GBM cells is a poor prognostic factor for tumors [10, 22, 23], the mechanism by which HS on the vascular endothelium affects GBM growth is unclear. Several studies have examined tumor growth in mice with endothelial cell-specific loss of HS. Fuster et al. [24] reported that, in endothelial cell-specific knockout mice of *Ndst1*, which encodes the HS *N*-acetylglucosamine *N*-deacetylase/*N*-sulfotransferase 1, the growth of lung cancer cells was significantly suppressed in the transplantation models. It has also been reported that HS 6-*O*-sulfation in the vascular endothelium is elevated in ovarian cancer, and that inhibition of HS 6-*O*-sulfotransferases 1 and 2 in ovarian cancer significantly suppressed tumor growth and angiogenesis in vivo [25, 26]. Similar to these reports, angiogenesis was significantly suppressed in our experiment, which may have greatly affected tumor growth (Fig. 5e). As mentioned in Secondary structure of Scherer [27], migration to perivascular area is occurred in glioblastoma invasion [28]. Therefore, decreased angiogenesis may partially suppressed glioblastoma invasion, leading to tumor growth reduction.

FGF2 is one of the most potent angiogenic stimulators in tumor growth and is known to be a stronger angiogenic factor than VEGF, i.e., the current therapeutic target in GBM [29, 30]. FGF2 is retained in the basement membrane in a stable state and secreted by astrocytes and GBM [31–34]. FGF2 has a strong angiogenic effect; however, the presence of HS is essential for the efficient expression of its angiogenic effect. A large difference in effect expression between HS derived from vascular endothelial cells and HS derived from the extracellular matrix has been reported [31]. In this study, only HS on the vascular endothelium was attenuated, but the effect of FGF2 was significantly reduced.

In our study, Ki67-postive tumor cells were not suppressed in *Ext1*^*CKO*^ group. Although reduced angiogenesis results in a hypoxic environment, Ki67 of glioblastoma is reported to not be suppressed despite of hypoxic condition in some articles like our result [35, 36]. On the other hand, Webster et al. suggested that tumor cell cycle become slowly in an ischemic environment, and some tumor cells rest in G2 phase permanently [37]. Thus, although the apparent proliferative capacity is maintained, it is possible that a decrease in proliferative capacity is actually observed. In addition, our result may be influenced by the fact that the sections were prepared at 14 days after tumor transplantation, which is a relatively early timing, leading to mild ischemia that glioma can somehow grow even slowly.

HS is one of the components of the glycocalyx in the BBB that is involved in maintaining homeostasis and regulating the inflammatory response [38–40]. Therefore, we investigated homeostasis and immunity but found no significant changes. This is probably because the glycocalyx in the brain is overwhelmingly thicker than other organs and is maintained to some extent even if damaged. It is also reported that chondroitin and dermatan sulfate are upregulated when heparan sulfate is reduced [41]. Therefore, it seems that the decrease in HS alone does not disrupt endothelial function [42].

The limitations of this study are that it is a transplant model and cannot imitate the natural course and that the effects on the immunity of organs other than the brain and immunity at the time of exacerbation of inflammation, such as infectious diseases, cannot be denied. It is also known that glioma may become malignant if the ischemic state continues, but long-term changes can’t be investigated in this model [43]. Further research is required to accurately assess the impact of these events.

## Conclusions

Specific reduction of HS in vascular endothelial cells suppresses tumor growth of GBM. Therefore, HS on the vascular endothelium has an important effect on angiogenesis in GBM.

## Supplementary Information

Additional file 1 Tab. S1 Primer details used for real-time RT-PCR (PDF 154 KB)

Additional file 2 Fig. S1 *Ext2* gene expression of endothelial cells isolated from the mouse brain. a. Quantification of relative expressions of *Ext2* in endothelial cells of the mouse brain evaluated by real time RT-PCR. (n=3 each cohort. Bars represent the mean ± SD. Student t test, n.s. = not significant). Fig. S2 Heparan Sulfate reduced blood vessels don’t regenerate so much in Matrigel assay. a. Appearance of tdTomato at Fig. 5c place of Matrigel plugs resected from Ext1 flox/flox; VE-Cre; Lsl-tdTomato and control (VE-Cre; Lsl-tdTomato) mice without FGF2 induction. Scale bar = 50 µm. Fig. S3 GL261 glioma appearance transplanted in murine brains. a-b. GL261 glioma appearance transplanted in murine brains of Ext1 flox/flox; VE-Cre and control (Ext1 flox/flox or Ext1 flox/+) mice. Schema (a) and Fig. 3a glioma’s H&E staining of every 200 µm as representative images (b). All brain images are adjusted to show the same side among images. Scale bar = 500 µm. Fig. S4 Immunostaining of pERK1/2 in tumor tissue is weakened with heparan sulfate reduction. a-b. Immunostaining of pERK1/2 (a) and ERK1/2 as control (b) in tumor tissues of Ext1 flox/flox; VE-Cre and control (Ext1 flox/flox or Ext1 flox/+) mice. Scale bar = 100 µm. Fig. S5 Cell density and nuclear atypia in tumor tissues are confirmed in DAPI images. a. Appearance of DAPI retaken at the same site of Fig. 4g. Scale bar = 50 µm (PDF 660 KB)

Additional file 3 (DOCX 17 KB)

## Data Availability

The datasets generated or analyzed during the current study are available from the corresponding author on reasonable request. All microarray data were deposited in Gene Expression Omnibus (GEO) under Accession No. GSE175419 (http://www.ncbi.nlm.nih.gov/geo/).

## References

[1] Yamada T, Tsuji S, Nakamura S, Egashira Y, Shimazawa M, Nakayama N, Yano H, Iwama T, Hara H (2020). Riluzole enhances the antitumor effects of temozolomide via suppression of MGMT expression in glioblastoma. J Neurosurg.

[2] Le Joncour V, Guichet PO, Dembele KP, Mutel A, Campisi D, Perzo N, Desrues L, Modzelewski R, Couraud PO, Honnorat J, Ferracci FX, Marguet F, Laquerriere A, Vera P, Bohn P, Langlois O, Morin F, Gandolfo P, Castel H (2021). Targeting the urotensin II/UT G protein-coupled receptor to counteract angiogenesis and mesenchymal hypoxia/necrosis in glioblastoma. Front Cell Dev Biol.

[3] Gao Y, Zheng H, Li L, Feng M, Chen X, Hao B, Lv Z, Zhou X, Cao Y (2021). Prostate-specific membrane antigen (PSMA) promotes angiogenesis of glioblastoma through interacting with ITGB4 and regulating NF-kappaB signaling pathway. Front Cell Dev Biol.

[4] Chinot OL, Wick W, Mason W, Henriksson R, Saran F, Nishikawa R, Carpentier AF, Hoang-Xuan K, Kavan P, Cernea D, Brandes AA, Hilton M, Abrey L, Cloughesy T (2014). Bevacizumab plus radiotherapy-temozolomide for newly diagnosed glioblastoma. N Engl J Med.

[5] Gilbert MR, Dignam JJ, Armstrong TS, Wefel JS, Blumenthal DT, Vogelbaum MA, Colman H, Chakravarti A, Pugh S, Won M, Jeraj R, Brown PD, Jaeckle KA, Schiff D, Stieber VW, Brachman DG, Werner-Wasik M, Tremont-Lukats IW, Sulman EP, Aldape KD, Curran WJ, Mehta MP (2014). A randomized trial of bevacizumab for newly diagnosed glioblastoma. N Engl J Med.

[6] Dallinga MG, Habani YI, Schimmel AWM, Dallinga-Thie GM, van Noorden CJF, Klaassen I, Schlingemann RO (2021). The role of heparan sulfate and neuropilin 2 in VEGFA signaling in human endothelial tip cells and non-tip cells during angiogenesis in vitro. Cells.

[7] Chua CC, Rahimi N, Forsten-Williams K, Nugent MA (2004). Heparan sulfate proteoglycans function as receptors for fibroblast growth factor-2 activation of extracellular signal-regulated kinases 1 and 2. Circ Res.

[8] Qiao D, Meyer K, Mundhenke C, Drew SA, Friedl A (2003). Heparan sulfate proteoglycans as regulators of fibroblast growth factor-2 signaling in brain endothelial cells. Specific role for glypican-1 in glioma angiogenesis. J Biol Chem.

[9] Loilome W, Joshi AD, ap Rhys CM, Piccirillo S, Vescovi AL, Gallia GL, Riggins GJ (2009). Glioblastoma cell growth is suppressed by disruption of fibroblast growth factor pathway signaling. J Neurooncol.

[10] Tran VM, Wade A, McKinney A, Chen K, Lindberg OR, Engler JR, Persson AI, Phillips JJ (2017). Heparan sulfate glycosaminoglycans in glioblastoma promote tumor invasion. Mol Cancer Res.

[11] Busse-Wicher M, Wicher KB, Kusche-Gullberg M (2014). The exostosin family: proteins with many functions. Matrix Biol.

[12] Inatani M, Irie F, Plump AS, Tessier-Lavigne M, Yamaguchi Y (2003). Mammalian brain morphogenesis and midline axon guidance require heparan sulfate. Science.

[13] Shimokawa K, Kimura-Yoshida C, Nagai N, Mukai K, Matsubara K, Watanabe H, Matsuda Y, Mochida K, Matsuo I (2011). Cell surface heparan sulfate chains regulate local reception of FGF signaling in the mouse embryo. Dev Cell.

[14] Kogata N, Arai Y, Pearson JT, Hashimoto K, Hidaka K, Koyama T, Somekawa S, Nakaoka Y, Ogawa M, Adams RH, Okada M, Mochizuki N (2006). Cardiac ischemia activates vascular endothelial cadherin promoter in both preexisting vascular cells and bone marrow cells involved in neovascularization. Circ Res.

[15] Kawai S, Takagi Y, Kaneko S, Kurosawa T (2011). Effect of three types of mixed anesthetic agents alternate to ketamine in mice. Exp Anim.

[16] Lind T, Tufaro F, McCormick C, Lindahl U, Lidholt K (1998). The putative tumor suppressors EXT1 and EXT2 are glycosyltransferases required for the biosynthesis of heparan sulfate. J Biol Chem.

[17] Lin X, Wei G, Shi Z, Dryer L, Esko JD, Wells DE, Matzuk MM (2000). Disruption of gastrulation and heparan sulfate biosynthesis in EXT1-deficient mice. Dev Biol.

[18] McCormick C, Leduc Y, Martindale D, Mattison K, Esford LE, Dyer AP, Tufaro F (1998). The putative tumour suppressor EXT1 alters the expression of cell-surface heparan sulfate. Nat Genet.

[19] Okada M, Nadanaka S, Shoji N, Tamura J, Kitagawa H (2010). Biosynthesis of heparan sulfate in EXT1-deficient cells. Biochem J.

[20] Forsberg E, Kjellen L (2001). Heparan sulfate: lessons from knockout mice. J Clin Invest.

[21] Gao H, Zhang IY, Zhang L, Song Y, Liu S, Ren H, Liu H, Zhou H, Su Y, Yang Y, Badie B (2018). S100B suppression alters polarization of infiltrating myeloid-derived cells in gliomas and inhibits tumor growth. Cancer Lett.

[22] Ohkawa Y, Wade A, Lindberg OR, Chen KY, Tran VM, Brown SJ, Kumar A, Kalita M, James CD, Phillips JJ (2021). Heparan sulfate synthesized by Ext1 regulates receptor tyrosine kinase signaling and promotes resistance to EGFR inhibitors in GBM. Mol Cancer Res.

[23] Kazanskaya GM, Tsidulko AY, Volkov AM, Kiselev RS, Suhovskih AV, Kobozev VV, Gaytan AS, Aidagulova SV, Krivoshapkin AL, Grigorieva EV (2018). Heparan sulfate accumulation and perlecan/HSPG2 up-regulation in tumour tissue predict low relapse-free survival for patients with glioblastoma. Histochem Cell Biol.

[24] Fuster MM, Wang L, Castagnola J, Sikora L, Reddi K, Lee PH, Radek KA, Schuksz M, Bishop JR, Gallo RL, Sriramarao P, Esko JD (2007). Genetic alteration of endothelial heparan sulfate selectively inhibits tumor angiogenesis. J Cell Biol.

[25] Ferreras C, Rushton G, Cole CL, Babur M, Telfer BA, van Kuppevelt TH, Gardiner JM, Williams KJ, Jayson GC, Avizienyte E (2012). Endothelial heparan sulfate 6-O-sulfation levels regulate angiogenic responses of endothelial cells to fibroblast growth factor 2 and vascular endothelial growth factor. J Biol Chem.

[26] Cole CL, Rushton G, Jayson GC, Avizienyte E (2014). Ovarian cancer cell heparan sulfate 6-O-sulfotransferases regulate an angiogenic program induced by heparin-binding epidermal growth factor (EGF)-like growth factor/EGF receptor signaling. J Biol Chem.

[27] Scherer H (1938). Structural development in gliomas. Am J Cancer.

[28] Hara A, Kanayama T, Noguchi K, Niwa A, Miyai M, Kawaguchi M, Ishida K, Hatano Y, Niwa M, Tomita H (2019). Treatment strategies based on histological targets against invasive and resistant glioblastoma. J Oncol.

[29] Birsner AE, Benny O, D'Amato RJ (2014). The corneal micropocket assay: a model of angiogenesis in the mouse eye. J Vis Exp.

[30] Cartland SP, Genner SW, Zahoor A, Kavurma MM (2016). Comparative evaluation of TRAIL, FGF-2 and VEGF-A-induced angiogenesis in vitro and in vivo. Int J Mol Sci.

[31] Elkin M, Ilan N, Ishai-Michaeli R, Friedmann Y, Papo O, Pecker I, Vlodavsky I (2001). Heparanase as mediator of angiogenesis: mode of action. FASEB J.

[32] Chadashvili T, Peterson DA (2006). Cytoarchitecture of fibroblast growth factor receptor 2 (FGFR-2) immunoreactivity in astrocytes of neurogenic and non-neurogenic regions of the young adult and aged rat brain. J Comp Neurol.

[33] Toyoda K, Tanaka K, Nakagawa S, Thuy DH, Ujifuku K, Kamada K, Hayashi K, Matsuo T, Nagata I, Niwa M (2013). Initial contact of glioblastoma cells with existing normal brain endothelial cells strengthen the barrier function via fibroblast growth factor 2 secretion: a new in vitro blood-brain barrier model. Cell Mol Neurobiol.

[34] Sooman L, Freyhult E, Jaiswal A, Navani S, Edqvist PH, Ponten F, Tchougounova E, Smits A, Elsir T, Gullbo J, Lennartsson J, Bergqvist M, Ekman S (2015). FGF2 as a potential prognostic biomarker for proneural glioma patients. Acta Oncol.

[35] Evans SM, Jenkins KW, Chen HI, Jenkins WT, Judy KD, Hwang WT, Lustig RA, Judkins AR, Grady MS, Hahn SM, Koch CJ (2010). The relationship among hypoxia, proliferation, and outcome in patients with de novo glioblastoma: a pilot study. Transl Oncol.

[36] Hatano T, Zhao S, Zhao Y, Nishijima K, Kuno N, Hanzawa H, Sakamoto T, Tamaki N, Kuge Y (2013). Biological characteristics of intratumoral [F-18]fluoromisonidazole distribution in a rodent model of glioma. Int J Oncol.

[37] Webster L, Hodgkiss RJ, Wilson GD (1998). Cell cycle distribution of hypoxia and progression of hypoxic tumour cells in vivo. Br J Cancer.

[38] Zhao F, Zhong L, Luo Y (2021). Endothelial glycocalyx as an important factor in composition of blood-brain barrier. CNS Neurosci Ther.

[39] Oshima K, King SI, McMurtry SA, Schmidt EP (2021). Endothelial heparan sulfate proteoglycans in sepsis: the role of the glycocalyx. Semin Thromb Hemost.

[40] Kutuzov N, Flyvbjerg H, Lauritzen M (2018). Contributions of the glycocalyx, endothelium, and extravascular compartment to the blood-brain barrier. Proc Natl Acad Sci U S A.

[41] Zaiss AK, Lawrence R, Elashoff D, Esko JD, Herschman HR (2011). Differential effects of murine and human factor X on adenovirus transduction via cell-surface heparan sulfate. J Biol Chem.

[42] Ando Y, Okada H, Takemura G, Suzuki K, Takada C, Tomita H, Zaikokuji R, Hotta Y, Miyazaki N, Yano H, Muraki I, Kuroda A, Fukuda H, Kawasaki Y, Okamoto H, Kawaguchi T, Watanabe T, Doi T, Yoshida T, Ushikoshi H, Yoshida S, Ogura S (2018). Brain-specific ultrastructure of capillary endothelial glycocalyx and its possible contribution for blood brain barrier. Sci Rep.

[43] Wen YD, Zhu XS, Li DJ, Zhao Q, Cheng Q, Peng Y (2021). Proteomics-based prognostic signature and nomogram construction of hypoxia microenvironment on deteriorating glioblastoma (GBM) pathogenesis. Sci Rep.

