# Supplementary material for: Endothelial cell-specific reduction of heparan sulfate suppresses glioma growth in mice
Source: Discov Oncol. 2021 Nov 11;12:50. doi: 10.1007/s12672-021-00444-3 (PMC8585801; doi:10.1007/s12672-021-00444-3)
Supplement: Supplementary file 1 — Additional file 1 Tab. S1 Primer details used for real-time RT-PCR (PDF 154 KB) [file 12672_2021_444_MOESM1_ESM.pdf]

FGF2	forward	5’-GCGACCCACACGTCAAACTA-3’
	reverse	5’-CCGTCCATCTTCCTTCATAGC-3’
VEGFA	forward	5’-CTTGTTCAGAGCGGAGAAAGC-3’
	reverse	5’-ACATCTGCAAGTACGTTCGTT-3’
EXT2	forward	5’-TGGGATCGAGGAACAAATCACC-3’
	reverse	5’-TGCCGGTAAGTCCAGGTAGAA-3’
β-actin	forward	5’-CATCCGTAAAGACCTCTATGCCAAC-3’
	reverse	5’-ATGGAGCCACCGATCCACA-3’
