# Supplementary figures and images for: Endothelial cell-specific reduction of heparan sulfate suppresses glioma growth in mice
Source: Discov Oncol. 2021 Nov 11;12:50. doi: 10.1007/s12672-021-00444-3 (PMC8585801; doi:10.1007/s12672-021-00444-3)

A

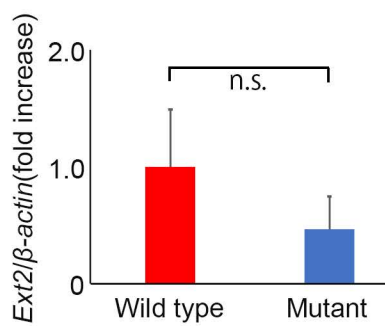

A

CD31 / tdTomato

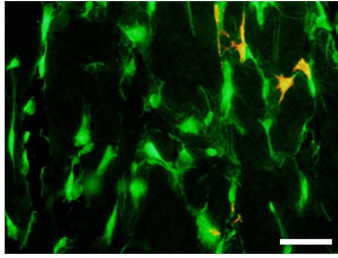

Wild type FGF2(-)

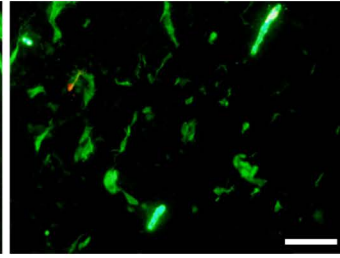

Mutant FGF2(-)

A

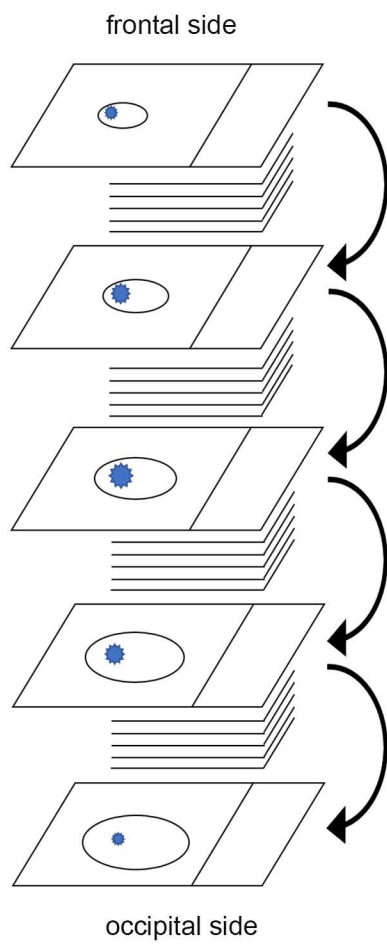

B

Wild type

Mutant

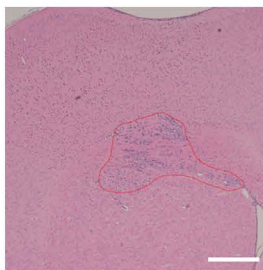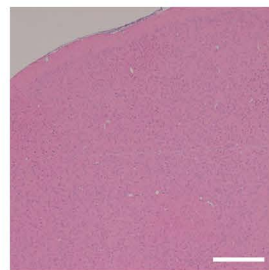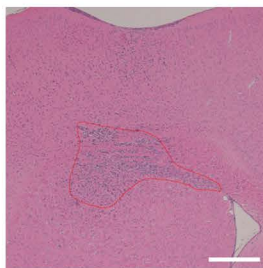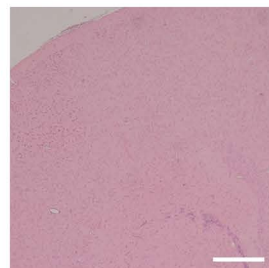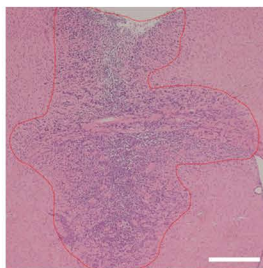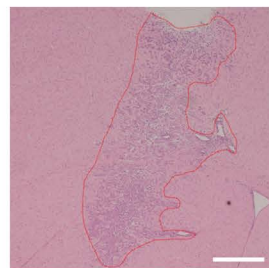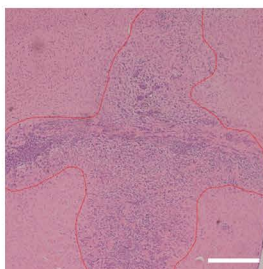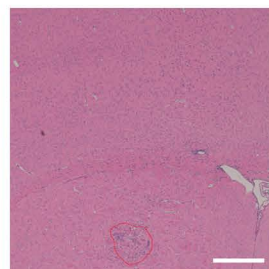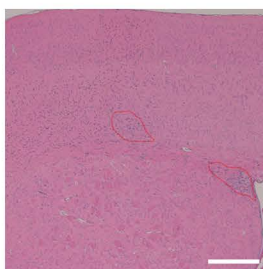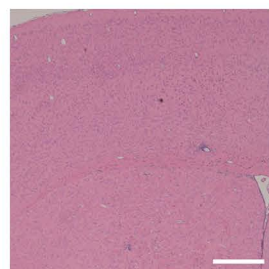

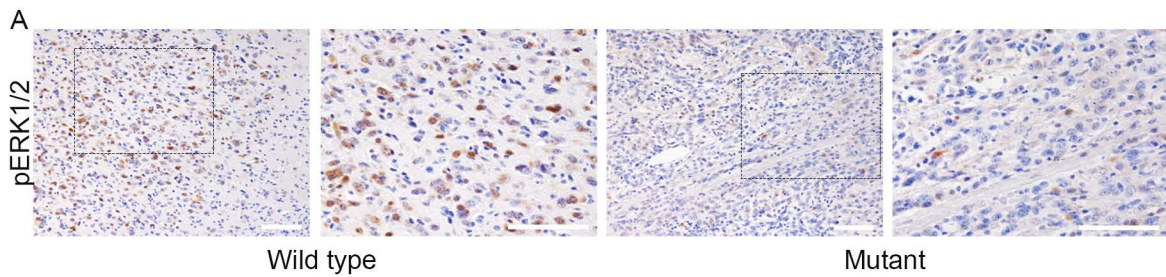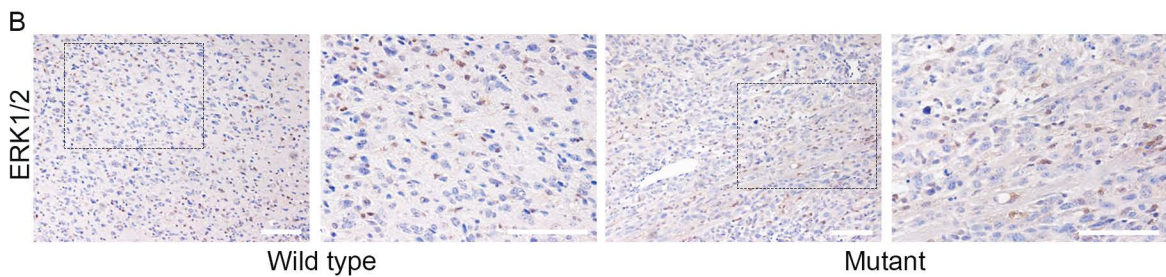

A

CD31/DAPI

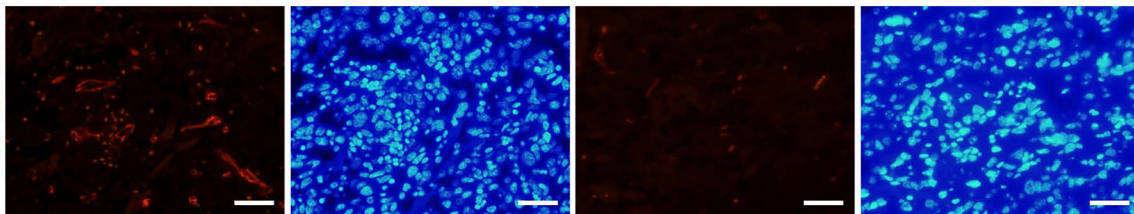

Wild type

Mutant

Supplement: Supplementary file 2 — Additional file 2 Fig. S1 Ext2 gene expression of endothelial cells isolated from the mouse brain. a. Quantification of relative expressions of Ext2 in endothelial cells of the mouse brain evaluated by real time RT-PCR. (n=3 each cohort. Bars represent the mean ± SD. Student t test, n.s. = not significant). Fig. S2 Heparan Sulfate reduced blood vessels don’t regenerate so much in Matrigel assay. a. Appearance of tdTomato at Fig. 5c place of Matrigel plugs resected from Ext1 flox/flox; VE-Cre; Lsl-tdTomato and control (VE-Cre; Lsl-tdTomato) mice without FGF2 induction. Scale bar = 50 µm. Fig. S3 GL261 glioma appearance transplanted in murine brains. a-b. GL261 glioma appearance transplanted in murine brains of Ext1 flox/flox; VE-Cre and control (Ext1 flox/flox or Ext1 flox/+) mice. Schema (a) and Fig. 3a glioma’s H&E staining of every 200 µm as representative images (b). All brain images are adjusted to show the same side among images. Scale bar = 500 µm. Fig. S4 Immunostaining of pERK1/2 in tumor tissue is weakened with heparan sulfate reduction. a-b. Immunostaining of pERK1/2 (a) and ERK1/2 as control (b) in tumor tissues of Ext1 flox/flox; VE-Cre and control (Ext1 flox/flox or Ext1 flox/+) mice. Scale bar = 100 µm. Fig. S5 Cell density and nuclear atypia in tumor tissues are confirmed in DAPI images. a. Appearance of DAPI retaken at the same site of Fig. 4g. Scale bar = 50 µm (PDF 660 KB) [file 12672_2021_444_MOESM2_ESM.pdf]
