# Supplementary material for: Endothelial cell-specific reduction of heparan sulfate suppresses glioma growth in mice
Source: Discov Oncol. 2021 Nov 11;12:50. doi: 10.1007/s12672-021-00444-3 (PMC8585801; doi:10.1007/s12672-021-00444-3)
Supplement: Supplementary file 3 — Additional file 3 (DOCX 17 KB) [file 12672_2021_444_MOESM3_ESM.docx]

**Histological evaluation**

***Immunostaining of cells***

Evaluation of immunostaining of cells was performed by using ImageJ/FIJI [1]. Briefly, the mean value of Ext1 or heparan sulfate (HS) inside the tdTomato positive area was measured in five areas of each cohort, and average value was calculated.

***Intensity of HS and dextran***

Intensity of HS and dextran on line scale was measured by using FV10-ASW 4.2 (Olympus).

***Histological and immunohistochemical procedures***

PDGFRβ was counted in five high power fields of each mouse, and average number was calculated. Ki67 and Iba1and CD31 were counted in hot spot, and average of listed number of mice was calculated. Ki67 and CD31 fluorescent staining were measured in high power field. Iba1 and CD31 immunostaining were counted in ×200 field. Ratio of positive area was evaluated by using ImageJ/FIJI.

***Matrigel angiogenesis assay***

The number of vessels was counted in five high power fields of each mouse, and average number was calculated.

1. Schindelin J, Arganda-Carreras I, Frise E, Kaynig V, Longair M, Pietzsch T, Preibisch S, Rueden C, Saalfeld S, Schmid B, Tinevez JY, White DJ, Hartenstein V, Eliceiri K, Tomancak P, Cardona A (2012) Fiji: an open-source platform for biological-image analysis. Nat Methods 9: 676-682 doi:10.1038/nmeth.2019
